# PI Prob: A risk prediction and clinical guidance system for evaluating patients with recurrent infections

**DOI:** 10.1371/journal.pone.0237285

**Published:** 2021-02-16

**Authors:** Nicholas L. Rider, Gina Cahill, Tina Motazedi, Lei Wei, Ashok Kurian, Lenora M. Noroski, Filiz O. Seeborg, Ivan K. Chinn, Kirk Roberts

**Affiliations:** 1 Department of Pediatrics, Baylor College of Medicine, Houston, Texas, United States of America; 2 Section of Immunology, Allergy and Retrovirology, Texas Children’s Hospital, Houston, Texas, United States of America; 3 Department of Information Services, Texas Children’s Hospital, Houston, Texas, United States of America; 4 Division of Allergy and Immunology, Massachusetts General Hospital, Boston, Massachusetts, United States of America; 5 The University of Texas School of Biomedical Informatics, Houston, Texas, United States of America; Vietnam National University, VIET NAM

## Abstract

**Background:**

Primary immunodeficiency diseases represent an expanding set of heterogeneous conditions which are difficult to recognize clinically. Diagnostic rates outside of the newborn period have not changed appreciably. This concern underscores a need for novel methods of disease detection.

**Objective:**

We built a Bayesian network to provide real-time risk assessment about primary immunodeficiency and to facilitate prescriptive analytics for initiating the most appropriate diagnostic work up. Our goal is to improve diagnostic rates for primary immunodeficiency and shorten time to diagnosis. We aimed to use readily available health record data and a small training dataset to prove utility in diagnosing patients with relatively rare features.

**Methods:**

We extracted data from the Texas Children’s Hospital electronic health record on a large population of primary immunodeficiency patients (n = 1762) and appropriately-matched set of controls (n = 1698). From the cohorts, clinically relevant prior probabilities were calculated enabling construction of a Bayesian network probabilistic model(PI Prob). Our model was constructed with clinical-immunology domain expertise, trained on a balanced cohort of 100 cases-controls and validated on an unseen balanced cohort of 150 cases-controls. Performance was measured by area under the receiver operator characteristic curve (AUROC). We also compared our network performance to classic machine learning model performance on the same dataset.

**Results:**

PI Prob was accurate in classifying immunodeficiency patients from controls (AUROC = 0.945; p<0.0001) at a risk threshold of ≥6%. Additionally, the model was 89% accurate for categorizing validation cohort members into appropriate International Union of Immunological Societies diagnostic categories. Our network outperformed 3 other machine learning models and provides superior transparency with a prescriptive output element.

**Conclusion:**

Artificial intelligence methods can classify risk for primary immunodeficiency and guide management. PI Prob enables accurate, objective decision making about risk and guides the user towards the appropriate diagnostic evaluation for patients with recurrent infections. Probabilistic models can be trained with small datasets underscoring their utility for rare disease detection given appropriate domain expertise for feature selection and network construction.

## Introduction

Infectious diseases contribute substantially to the costs of healthcare. For invasive Streptococcus pneumoniae alone, one estimate suggests that $3.5 billion US Dollars (USD) are spent in direct costs for complications related to this single organism [1]. Among individuals who suffer infectious disease, a subsection will have ongoing risk of morbidity and mortality owing to an underlying genetic susceptibility. Such patients with primary immune defects (PI) often present in subtle fashion and may be mistaken for individuals with routine infection leading to diagnostic delay and death [2].

Once thought to be rare, there are now 416 distinct PI diseases with 430 molecular etiologies known [3]. However, due to improved understanding about its biology and expanded testing capabilities, PI is now estimated to affect between 1:1000 to 1:5000 individuals and prior work suggest that upwards of 1% of the general population may have features of PI risk [4–6]. Yet, early recognition of PI remains a challenge with time to diagnosis remaining largely fixed over the past 4 decades and is estimated at 7.5–9 years from symptom onset [2]. Projected annual costs for PI patients prior to diagnosis is nearly $140,000 USD which may be reduced by approximately $78,000 USD following diagnosis [7,8]. In order to spare costs and undue morbidity, efforts are underway to facilitate early PI diagnosis and treat affected patients in a precise manner [4,9].

To aid in disease diagnosis, artificial intelligence methods can assist in population-wide and individual-level risk assessment [10,11]. In particular, models which meld reasoning and uncertainty can facilitate diagnosis amongst at-risk individuals [12]. Bayesian networks (BNs) are one such model which combine relevant features, joint probability distributions and an intuitive structure to answer questions in biomedicine [13]. Specifically, BNs have proven useful for clinical decision support for detecting lung cancers, prediction of heart failure, computing survival for individuals with colon cancer, aiding in liver disease evaluation and enabling pathology specimen evaluation among other use cases [6,14–19]. They are also particularly effective for generating predictions even when trained on small datasets [20]. It is for these reasons that we chose to build a BN for PI risk prediction and clinical guidance.

**Fig 1:**
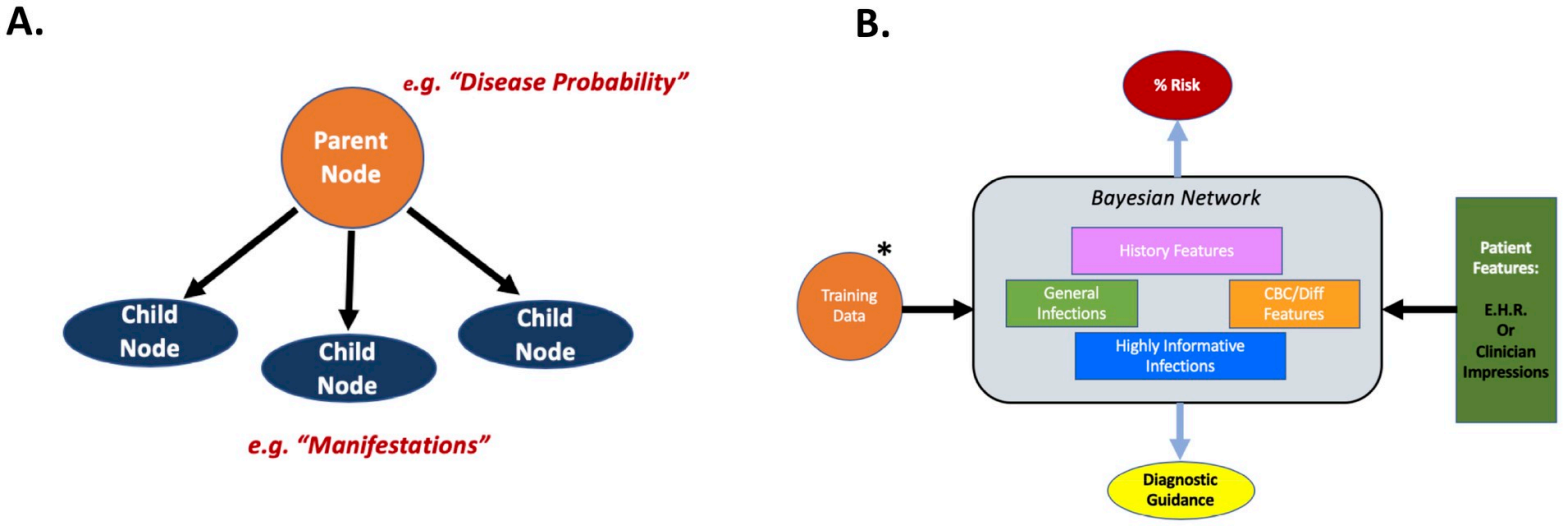
Bayesian network structure. **A**. An example Bayesian network with parent and child nodes connected by arcs. **B**. The general structure of our network components. Training data (*may be updated over time) and patient features allow for improving inference (i.e. probabilities) and structure over time making these networks dynamic. (EHR = Electronic Health Record).

More specifically, BNs are probabilistic graphical models which embed data, expert opinion or a combination of the two into an intuitive graph thereby enabling reasoning given inherent uncertainty [21]. The basic structure of a BN (Fig 1A) consists of nodes, each representing a variable, and arcs (arrows) which connect nodes in a causal relationship [22]. Underlying this intuitive and transparent structure are Bayes’ theorem and Bayesian statistics which allow for calculating conditional and joint probability distributions across many variables within the network [23,24]. The BN allows for both data and domain knowledge to be packaged into a single model that can yield insights about real-world probabilities, can be updated with new data or opinion over time and can tolerate missing information [13,21,22].

Here, we describe a BN which both assesses risk of underlying primary immunodeficiency and provides clinical guidance (Fig 1B). Our network model (PI Prob) was constructed by an expert immunologist but the parameters (i.e. conditional probabilities) were learned entirely from readily available electronic health record (EHR) data. PI Prob may be embedded within an EHR via an application programming interface (API) for the purposes of analyzing large-scale data in real time; alternatively, it may be clinician facing for data entry at the point of care to assess a given patient’s risk and guide diagnosis.

## Methods

### Ethics statement

This study was approved by the Baylor College of Medicine Institutional Review Board (H-38501). All patient data remained confidential and was retained in the Texas Children’s Hospital system.

### Cohort analysis

Patients from Texas Children’s Hospital (TCH) with immunodeficiency were identified by having at least 2 ICD10 codes (ICDCs) entered at different time points (2008–2018) which was consistent with a primary immunodeficiency as categorized by the American Academy of Asthma, Allergy and Immunology (AAAAI; https://www.aaaai.org/Aaaai/media/MediaLibrary/PDF%20Documents/Practice%20Management/finances-coding/ICD-10-Codes-Immunodeficiencies.pdf). The PI cohort was comprised of 1762 individuals (Table 1). In contrast, control data were specified by an age matched set of patients from TCH who did not harbor one of the PI codes and consisted of 1698 individuals. This was termed the “Control Cohort”. For each cohort, all ICDCs over 24 months were extracted from the TCH EHR (Epic Clarity Database). Individual, unique ICDCs were compiled for each cohort, counted, normalized and assessed for differences between groups. We focused on ICDCs which had a significantly increased frequency among PI patients and associated relevance to PI based upon domain expertise. Features for construction of the BN were derived from this list of PI cohort enriched ICDCs based upon their relevance to human immunological disease as determined by an expert clinical immunologist (NLR) (S1 Fig).

**Table 1:** Demographic features of the cohort.

	PI Cohort (n = 1762)	Control Cohort (n = 1698)
Mean Age (yrs)	8 ± 4	7 ± 5
Sex		
Male	1001 (56.8%)	891 (52.5%)
Female	761 (43.2%)	807 (47.5%)
Ethnicity:		
Caucasian	793 (45%)	552 (33%)
Hispanic	606 (35%)	601 (35%)
Black	199 (11%)	281 (17%)
Asian	89 (5%)	91 (5%)
Unknown	75 (4%)	173 (10%)

### Prior probability calculations

We calculated prior probabilities for 36 different feature nodes for both the PI and Control populations. Each feature had corresponding ICDCs as listed in Table 2 and was representative of the node concept [25]. Prior probabilities were calculated by taking the total number of occurrences for each ICDC of interest and dividing by the total number of patients in that specific cohort (either PI or Control). These conditional probabilities (i.e. fraction of individuals with the condition given PI or given non-PI) were then used to create the Bayesian Network as described below. When there were no occurrences of a given clinical entity among controls, we arbitrarily set the conditional probability to be 0.0001% since a prior value of “0%” or “100%” does not accurately reflect real-world uncertainty. Learning prior probabilities for the “meningococcal disease” node general risk differed from the rest in that it was derived from literature rather than from data; additionally, data about patients receiving complement inhibitor therapy were used as a surrogate for meningococcal disease risk in PI [26,27].

**Table 2:** Top 20 network features ranked by weight.

Rank	Node Description (ICD Code(s))	Count PI Cohort (No.)	Conditional Probability PI Cohort (%)	Count Control Cohort (No.)	Conditional Probability Control Cohort (%)	Weight**
1	Systemic Lupus Erythematosus	39	2.2	0	0.0001	22000
2	Eosinophilia (D72.1)	35	2.0	0	0.0001	19875
3	Auto-Immune Hemolytic Anemia (D59.1)	32	1.8	0	0.0001	18171
4	Meningococcal Disease (A39.xx)*	N/A	0.0001	N/A	0.0	1000
5	Lymphopenia (D72.810)	113	6.4	3	0.2	36
6	Hypocalcemia (E83.51)	49	2.8	3	0.2	16
7	Superficial Mycosis	16	0.9	1	0.06	15
8	Neutropenia (D70)	246	14.0	18	1.1	13
9	Opportunistic Infections (A02, A31,B39, B55, B40, B59)	26	1.5	2	0.1	13
10	Polyarthritis (M00, M13)	13	0.7	1	0.06	12
11	Sepsis (A40, A41, O85, P36)	104	5.9	9	0.5	11
12	Lymphoma (C81, C82, C83, C84, C85, C86, C88)	23	1.3	2	0.1	11
13	Thrombocytopenia (D69.59, T45.IX5A)	75	4.3	7	0.4	10
14	Septic Shock (A41.9, R65.21)	95	5.4	9	0.5	10
15	Recurrent Fever (A68)	30	1.7	3	0.2	9
16	Herpes Infections (A60, B00, B02, B10, B27)	100	5.7	15	0.9	6
17	Encephalitis (A83, A84, A85, A86, G04)	10	0.6	2	0.1	6
18	Fibrosis (K74)	6	0.3	1	0.06	6
19	Organomegaly (R16)	50	2.80	8	0.5	6
20	Mycobacterial Disease (A31.9)	10	0.6	2	0.1	5

* Denotes data learned from literature.

** Ratio of PI/Control Conditional Probability.

### PI Prob network construction

Network structure was designed to facilitate clinical decision making derived from information obtained from a thorough history/physical examination and complete blood count (CBC/differential) without need for advanced immunological testing. The BN described was constructed using GeNIe Modeler from BayesFusion, LLC (http://www.baysefusion.com/). Feature nodes (i.e. infections, lab findings, historical features) were used to build the network and were singly connected to the risk node since all features were deemed to be relevant for risk of PI. In total 36 feature nodes were used (10 history/physical examination, 5 laboratory, 5 general infections, 16 highly informative infection/condition) which corresponded to 79 distinct ICDCs (Fig 1B, S1 Fig & Table 2). Contained within each node are the conditional probabilities (i.e. prevalence) of finding that variable in the PI cohort (“med-high risk”) and within a general pediatric population (“low risk”). At baseline, the network displays the background prevalence of each node condition within the general population but selecting its presence for a given patient results in the instantiation of the prevalence we calculated from within our PI cohort. For example, abscess has a conditional probability of 1.5% in the control population and 3.7% in the PI cohort. The former represents the contribution to baseline risk in any patient a priori; whereas, presence of an abscess in the clinical history of a patient would contribute the corresponding conditional probability learned from the PI cohort (i.e. 3.7%). We assumed the general population overall *risk* to be 1% for PI. This threshold was determined experimentally from our prior work where 2188 of 185,892 individuals (1.2%) were deemed at medium-high risk for PI [4].

The PI Prob structure was designed such that all 36 diagnostic variables/features are connected to the risk node. Risk is then calculated by employing Bayes’ Theorem (Eq 1; *P(A|B) = probability of A given B*, *P(B|A) = probability of B given A*, *P(A = the probability of A occurring*, *P(B) = the probability of B occurring*) for single or multiple conditions as features are instantiated for a given patient. All features instantiated as “present” for a given patient, result in calculation of a joint probability across the entire distribution of present features according to Eq 2 (*similar to* Eq 1 *but here multiple independent events “B” are included enabling a calculation of the probability of A occurring given B*_*1*_ *to B*_*n*_) with the assumption of conditional independence. If unselected, the default joint probability is that of the general population risk.

$$P(A|B)=\frac{P(BA)*P(A)}{P(B)}$$ (1)

$$P(A|B1,\ldots ,Bn)=\frac{P(B1,\ldots ,BnA)*P(A)}{P(B1,\ldots ,Bn)}$$ (2)

Feature nodes were also connected via arrows (edges) to each of 8 IUIS disorder categories described as: Tcell/Combined (T/CID), Predominantly Antibody (PAD), Immune Dysregulatory (PIRD), Phagocyte (PD), Innate (ID), Autoinflammatory (AID) and Complement (CD). We combined IUIS tables 1 & 2 from the Expert Committee report into one category and did not include bone marrow failure syndromes or PI phenocopies. This resulted in 7 IUIS disorder network nodes. Node connections were created based upon their contribution to a phenotype for that category as expected by domain expertise or as described in Tangye et al [3] (S1 Fig). Arrow directions indicate dependency (e.g. moving in the direction of the arrow one can say “*this* is dependent upon *that*”). For a given patient, IUIS category likelihood ranking was calculated by summing individual conditional probabilities among the present feature nodes. The output IUIS categories with the highest “yes” probability suggest the most appropriate starting point in the diagnostic workup for an individual patient. The probability output shown is compared to the general population; therefore, magnitude change from baseline is more important than the absolute percentage shown.

### PI Prob training and validation

**Fig 2:**
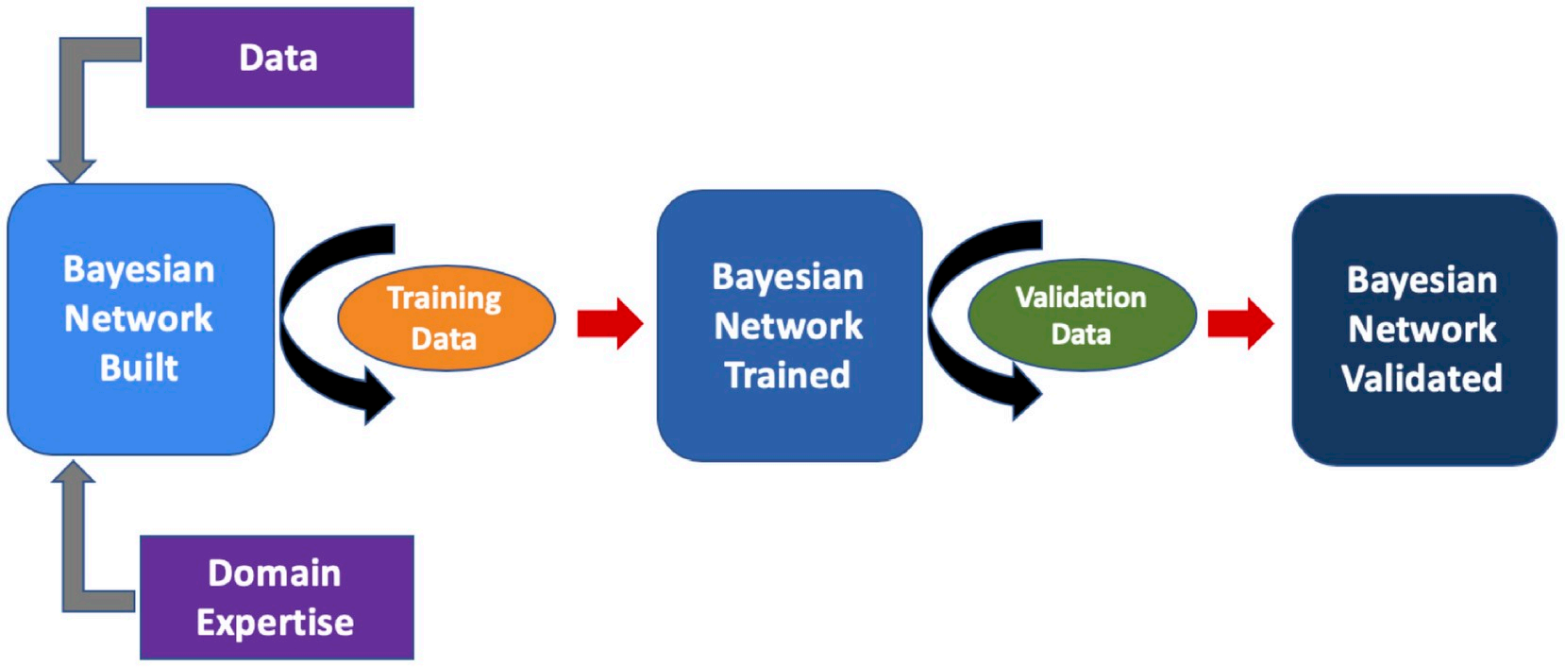
Analytics plan. Network construction, training and validation scheme. Here, 100 patients (50 PI and 50 Control) were used as training data and 150 (75 each cohort) were used as validation data.

From our immunodeficient and control cohorts, 250 patients (125 disease, 125 control) were randomly selected to train and validate the network. There was no overlap between PI and control data; similarly, patient data used for training was distinct from that used to validate the network. For the training and validation cohorts, each patient’s clinical and relevant laboratory history was confirmed by medical record chart review. Additionally, we assessed SDOH via analysis of insurance type as a proxy to normalize for health care access across the cohorts. Features were determined and entered into the BN for calculation of risk and IUIS category ranking (Fig 2). For PI cohort training and validation, we excluded patients with a diagnosis of severe combined immunodeficiency (SCID) since they should have been detected asymptomatically via newborn screening. Similarly, patients with secondary immunodeficiency were excluded from the PI group but not from the control group.

**Fig 3:**
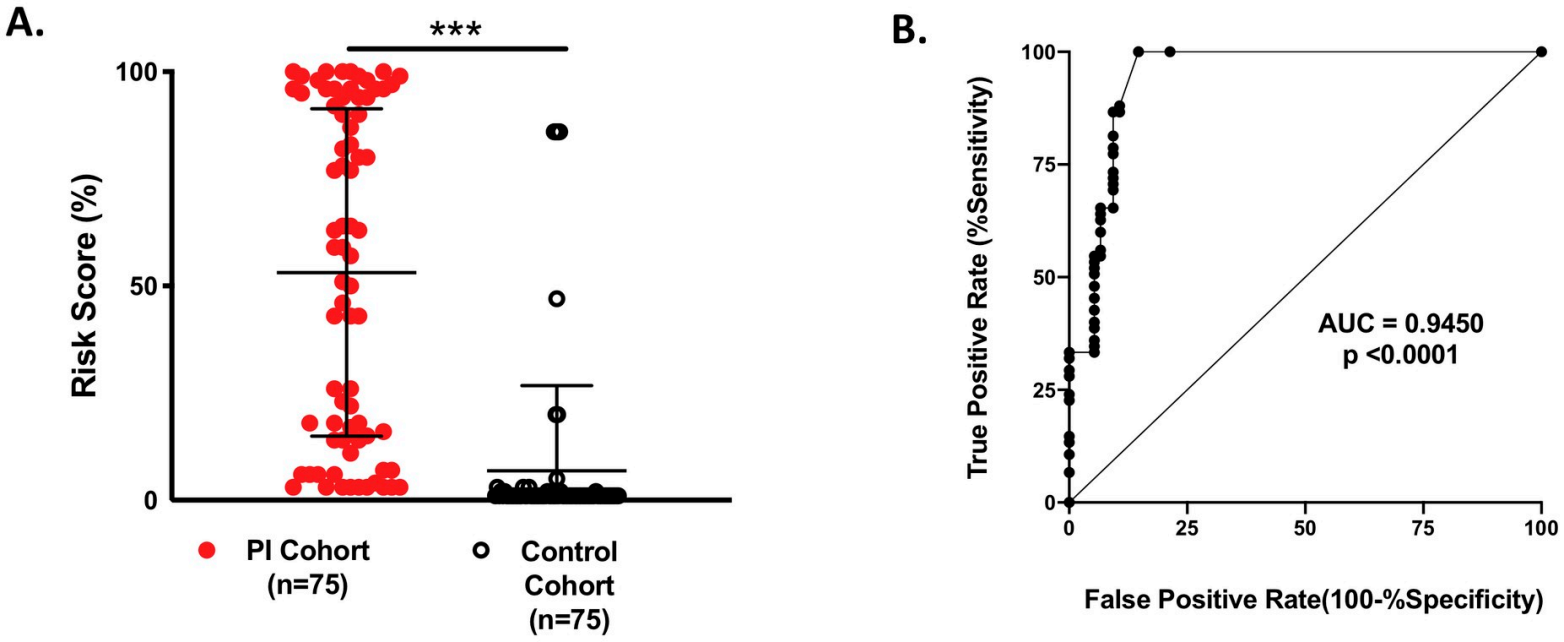
Network validation. Validity testing of our BN for individual patients from the PI and Control cohorts. **A**. Mean risk scores between the two populations were significantly different (53% vs. 7%; p <0.000001). **B**. Network performance as calculated by AUROC (Area under Receiver Operator Characteristic Curve) where an AUROC of 1.0 represents the ability of a model to discriminate between classes 100% of the time.

After initial model build (i.e. node selection, probability embedding and arc-setting), we trained our BN with 50 PI patients and 50 controls (“training data”; Fig 2). This allowed for tuning of the BN, adding additional relevant features and provided insights about node connections. Once the network was restructured, we performed validation testing with 75 previously unseen patients from each cohort (“validation data”; Fig 3). Members of the validation cohorts had similar ages (mean control age = 8 ± 5yrs; mean PI age = 8 ± 3 years). Network performance was assessed by receiver operator characteristic (ROC) curve analysis on the validation cohort.

### Comparison to other machine learning models

Using the same PI and Control cohorts as described above, we trained three additional models (logistic regression, Naïve Bayes and support vector machine (SVM) classifiers) using scikit-learn (v 0.23; https://scikit-learn.org/stable/). The models were then serialized and validated using the identical 75 PI patients and 75 controls as described above for PI Prob validation (https://github.com/nlrider/PI-Prob).

### Statistical methods

Descriptive statistics for the PI and control cohorts were determined using Microsoft Excel and are shown in Table 1. Significance testing and the ROC curve analyses were conducted in Prism GraphPad version 8 (https://www.graphpad.com). Comparison machine learning model validation performance was performed in scikit-learn to calculate receiver operator area under curve (AUROC), F1 scores, precision and recall.

## Results

### PI cohort vs. control cohort

Demographic statistics for the PI and control populations are shown in Table 1. The proxy SDOH and healthcare access via assessment of insurance coverage and type was similar between groups (PI Cohort: 52% Private, 45% Public, 3% Unknown; Control Cohort: 48% Private, 50% Public, 2% Unknown). Network feature nodes are shown in Table 2 and were all significantly different in the PI vs. control cohorts (p-value <0.0001). Table 2 shows the top 20 features in our network ranked by relative conditional probability comparison between the PI and Control cohorts.

### Risk calculation (PI cohort vs. controls)

Performance of the BN on our validation cohort is shown in Fig 3. Individual risk calculation is displayed for the immunodeficient and control patients (Fig 3A) as a single assessment for a given patient at the time of chart review. Classification performance is shown in Fig 3B and displayed as a receiver operator characteristic curve (ROC). The ROC analysis predicted best model performance at a risk cutoff of >5.5% (Sensitivity = 87%; CI (77%-93%) and Specificity = 91%; CI (82%-96%)). The corresponding BN performance measures were subsequently calculated as reported in Table 3 for risk calculation >5.5%.

**Table 3:** Model performance comparisons.

Model	Precision	Recall	F1 Score	AUC
PI Prob (Bayesian Network)	0.90	0.87	0.88	0.95
Logistic Regression Classifier (LogReg)	0.99	0.81	0.89	0.90
Naïve Bayes Classifier (NBC)	1.00	0.80	0.89	N/A
Support Vector Machine (SVM)	0.99	0.81	0.89	0.90

AUC = Receiver Operator Area Under Curve.

### Comparison of models

Three classic machine-learning (ML) models were trained and validated for comparison to PI Prob. The performance testing is shown in Table 3. Model performance was similar across all 4 models validated. PI Prob displayed the greatest AUC and the Naïve Bayes Classifier displayed overfitting with an inability to discriminate true negatives; thus, an AUC was not calculable.

### Prescriptive output—Directing clinical management

**Fig 4:**
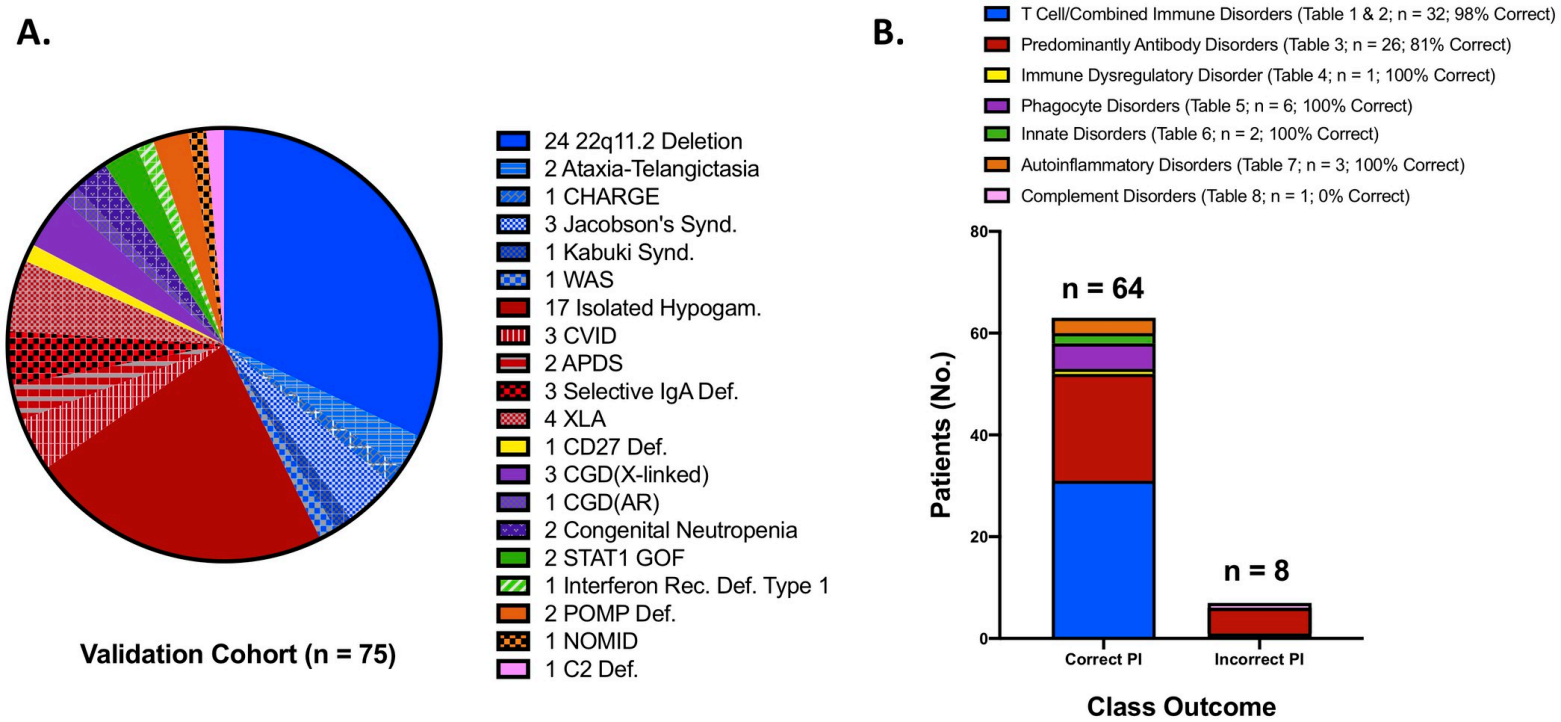
Cohort features & network outcomes. A. Validation cohort disorder spectrum. The IUIS groupings are clustered according to color (i.e. Blue = T/CID; Red = PAD; Yellow = PIRD; Purple = PD; Green = ID; Orange = AID and Pink = CD. B. BN performance for classifying each IUIS category and overall outcome. The legend displays category number and accuracy for our BN prediction. NOTE- 3 patients were not included here since insufficient input data were available and a class outcome could not be defined by the model. (Abbreviations: CHARGE-coloboma, heart disease, atresia of choanae, restricted growth, genital and ear abnormalities; WAS-Wiskott-Aldrich Syndrome; CVID-Common Variable Immunodeficiency; APDS-Activated Pi3K Delta Syndrome; XLA-X-linked Agammaglobulinemia; CGD-Chronic Granulomatous Disease; STAT1 GOF- Signal Transducer Activator of Transcription 1 Gain of Function; POMP-Proteasome Maturation Protein; NOMID-Neonatal Onset Multisystem Inflammatory Disease).

Individuals within the PI validation cohort (n = 75) had 20 distinct PI disorders spanning the first 8 IUIS category tables (Fig 4A). Within the validation cohort, most had an IUIS table 1/table 2 (T/CID) related disorder (n = 32; 43%) or an IUIS table 3 (PAD) related disorder (n = 26; 35%). Of the remaining disease categories, IUIS table 4 (PIRD) comprised 1% (n = 1), IUIS table 5 (PD) comprised 8% (n = 6), IUIS table 6 (ID) comprised 3% (n = 2), IUIS table 7 (AID) comprised 4% (n = 3) and IUIS table 8 (CD) comprised 1% (n = 1).

PI Prob performance for predicting each patient’s top 2 most likely IUIS disease categories is shown in Fig 4B. The BN was most successful in diagnosing patients with T/CID and phagocytic disorders. It was moderately effective in classifying antibody deficient patients appropriately. Additionally, the BN accurately classified patients with immune dysregulation, innate disease and autoinflammatory disease, but failed to classify the one complement disorder patient in our cohort. Overall BN model accuracy for determining patients across any studied IUIS category was 89%. It was 86% accurate if patients with IUIS table 1–3 disorders were omitted (n = 17).

### Bayesian network functionality and usability

**Fig 5:**
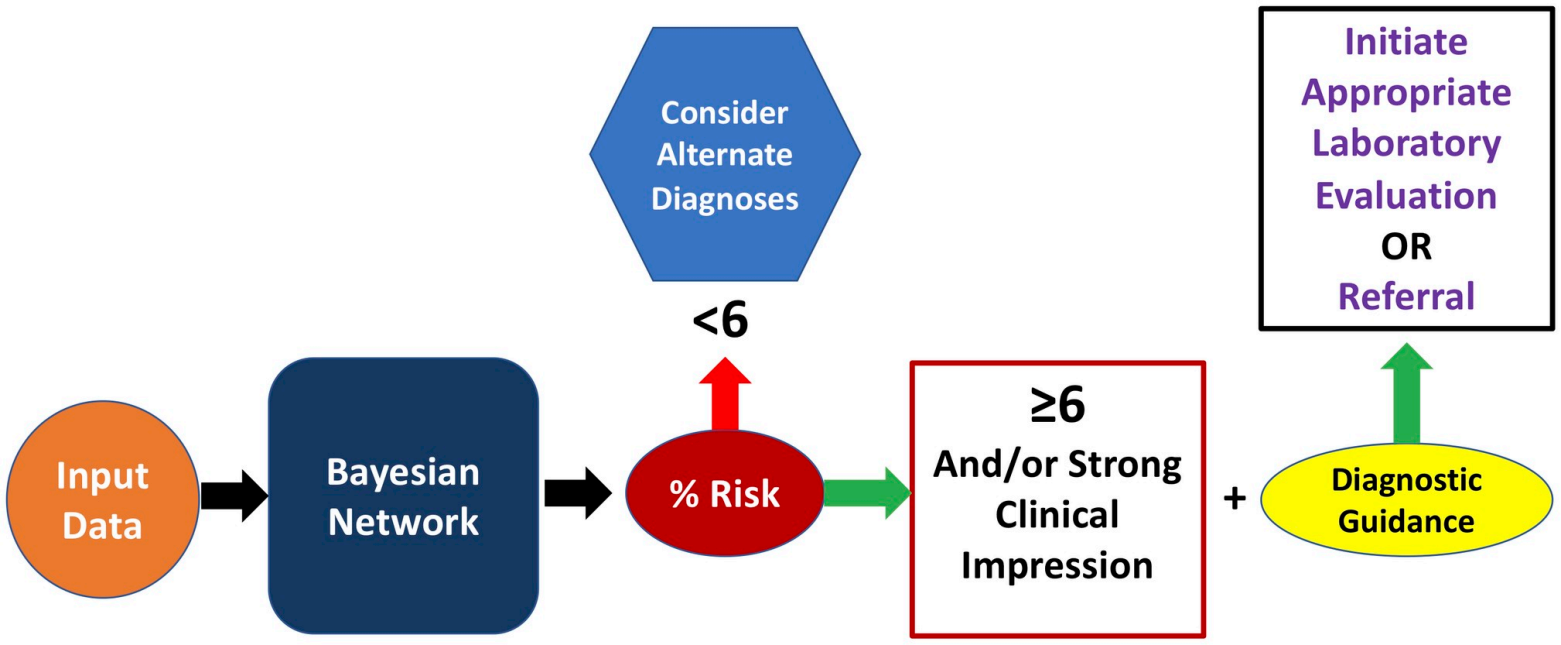
Workflow model. The proposed workflow for our model. Here, an end-user or EHR data feed can provide inputs via clinical impressions or diagnostic codes. The BN calculates a risk score which can subsequently be acted upon. It is important to note that it is the risk score and clinical impression should be taken together, which guide subsequent evaluation and management.

Data flow though the BN occurs once patient information is instantiated by an end-user or by analyzing diagnostic codes by EHR automatic feed via an API (S1B & S1C Fig). The native structure is shown in Panel A; however, one can see the outputs for “Risk” and IUIS Category change depending upon data input. As displayed in S1B.1 & S1B.2 Fig a patient’s data with X-linked agammaglobulinemia (XLA) and associated clinical features are instantiated. The BN then provides an updated risk prediction and a prescription about the most likely diagnostic category (i.e. PAD) which can then inform the initial immunological workup S1C.1 & S1C.2 Fig shows information flow for a patient with chronic granulomatous disease (CGD) whose clinical findings are instantiated. Here risk is again updated from baseline and the prescriptive component selected PD as the top predicted IUIS category. A schematic of the proposed workflow is shown in Fig 5.

## Discussion

Knowledge about the biology of PI is expanding rapidly; however, overall diagnostic rates have not appreciably improved [2,3,5]. Therefore, novel disease-detection methods are needed such as digitizing relevant phenotypes and leveraging health information technology functionality. Here we demonstrate that concepts related to PI can be learned from readily available, EHR-mined, structured data and embedded within a transparent (Fig 1B) machine learning model that accurately provides both risk assessment and clinical guidance (i.e. ICDCs; Table 2, Fig 2). The model, a BN, allows for concept linking driven by domain expertise thereby enabling information flow in a rational manner for disease detection [6,14,28,29]. This model can be deployed in almost any clinical setting and may learn over time as population specific conditional probabilities are accumulated with ongoing accuracy refinement [30,31].

PI Prob performed well on an unseen validation cohort of 75 patients with PI possessing a diverse range of diseases across the IUIS spectrum with similar performance to the other ML models built (Table 3, Figs 3 and 4). The AUROC (0.945; p < 0.0001, Fig 3B) suggests robust model classification of PI patients vs. control individuals and less susceptibility to overfitting. Training of the model consisted of analyzing only 50 PI patients and 50 controls to assess performance which allowed us to identify gaps in concept relationships (Fig 2). The small training set suggests utility of probabilistic graphical models in the rare disease space. Fig 3A shows considerable scatter across risk scores for the PI validation cohort; however, the mean group score was significantly different from that of the controls (53.1±38.1 vs. 6.9±20; p < 0.000001) providing evidence that our model’s inference about PI is sound. Importantly, our age-matched and SDOH-similar control group consisted of patients with varying medical complexity themselves but not PI. Diagnoses among this group included cystic fibrosis, trisomy 21, acute lymphoblastic leukemia, solid organ cancers, asthma, complex congenital heart disease and complex genetic disease. Therefore, accurately distinguishing PI from our controls is likely a more challenging task than classification against the general pediatric population. We take this as evidence of real-world model fitness and utility for our BN.

In addition to risk prediction, PI Prob provides a prescriptive outcome to facilitate appropriate diagnostic evaluations and initiate referral if needed (Figs 4 and 5). The BN’s ability to direct a diagnostic approach is two-fold (Fig 5). First, a clinician’s threshold for testing should be triggered by their clinical impression and a risk score ≥6%. The next question, “what do I do now?”, can be answered via guidance about the most likely IUIS category. Given noted phenotypic heterogeneity among PI disorders, we assumed the initial predicted IUIS category would not be sufficiently inclusive; thus, we built our BN to predict the top 2 IUIS categories for each PI patient [32]. Using this strategy, our model was able to accurately define this class 89% of the time for the PI validation cohort (Fig 4). In 3 cases, the BN was unable to provide clinical guidance owing to lack of sufficient information. This scenario results in an equal probability prediction across IUIS categories and underscores the diagnostic uncertainty when scant patient information is available.

The prescriptive output of PI Prob has utility for the primary care provider and clinical immunologist. Our expectation is that the provided decision guidance will inform clinical encounters by expediting appropriate testing and accurate diagnosis, assuming availability of recommended immune-diagnostic testing [33]. For the generalist or clinicians who are not generally focused on PI patients, we envision enabling early initial testing and referral to improve diagnostic rates. For the expert clinical immunologist, having relevant results in hand at the time of initial encounter should facilitate early implementation of best treatment practices and drive optimal outcomes for patients.

From an epidemiologic standpoint, PI Prob’s best predicted risk score cutoff of ≥6% is interesting in that it aligns well with our previous work in calculating a risk vital sign for PI [4]. There, analyzing a different population, we found 1% of the general population to be at medium-high risk for PI and subsequently ~5% of this group had PI or a concerning infectious diagnosis in the following year [4]. These results suggest that the prior probability of disease (prevalence) approaches 5% for individuals deemed to be of medium-high risk. Thus, all healthcare providers may expect approximately 1% of their patients to be at risk for PI. These individuals must then be distinguished from individuals with actual disease further reinforcing the importance of considering this vulnerable patient population amongst anyone with infections.

Here, we hypothesize that clinical informatics and AI methods may inform the diagnostic process by combining disease-specific features and epidemiologic data to enable diagnosis. Such models require computational transparency, good performance and should extend optimal digital health workflows to align with clinical decision support (CDS) best practices [34]. Predictive analytics allows for discrimination about what might happen in a given clinical scenario; whereas, prescriptive analytics focuses on what one should do about the prediction [35]. This BN packages these attributes and presents a dual output for each patient to the user. Given reported concerns about time pressure for healthcare providers, delays in diagnosis, increased costs and poor outcomes we hoped to address these obstacles with our model en route to lowering the bar for PI diagnosis [2,7,36]. Lastly, we wish to underscore that our view of AI’s role in healthcare is that it should be rigorously validated but dutifully implemented to augment clinical decision making and extend clinical efforts for making use of big data in a way that serves the patient and provider [37,38]. With this perspective AI/machine learning aids the clinician and makes health information technology more useful.

## Limitations and future directions

While our model performed very well with the validation cohort, we need to test it prospectively on larger numbers of patients. Also, it will be helpful to have a larger number of end-users provide feedback via a web-interface to improve usability (S1 Fig). Future work in this regard will focus on expanded testing and soliciting expert immunologist opinions broadly as they use the BN for patient assessments.

Another limitation of our study was the somewhat biased PI cohort with a large number of 22q11.2 deletion syndrome patients (n = 24). The model was very accurate in predicting their IUIS class, but some of these individuals may have been detected via TREC-based newborn screening. This bias is reflected in our overall PI population which contained many DiGeorge Syndrome patients cared for at our center. Additionally, we had limited representation of patients with disorders falling into the IUIS tables 4, 6, 7 and 8. Patients with these disorders are less prevalent among PI as a whole; however, such information could be mined from registries or pooled from other centers and used to improve model fitness. Also, we decided to exclude inference about marrow failure syndrome and PI phenocopies (IUIS tables 9 & 10) here; thus, patients with these disorders might not be detected with the current version of our network. We can track model performance prospectively about patients with such disorders and easily modify the network as needed.

Lastly, we plan to investigate additional variables that can be added to further improve network performance. Our BN performs very well in validation with only 36 nodes; however, it is likely that additional informative features will drive further improvement.

## Conclusions

Use of this BN and other machine learning approaches, may facilitate diagnosis of patients with PI. Combining domain expertise and readily available EHR structured data with well-defined machine learning models provides an effective tool for risk assessment of PI. PI Prob is interoperable and demonstrates the utility of using probabilistic graphical models for improving rare disease detection given their favorable performance when trained with relatively small datasets.

## Supporting information

S1 Fig Network structure and example web interface. A.1 The native BN structure and low-risk probabilities specified are shown. Users can see background probabilities for low/high risk and select nodes here if desired. A.2 Native dashboard interface. Users can select features along the right sidebar and see risk and top diagnosis prediction displayed on the left panels. B.1 A case example of network information flow upon entering clinical data for a patient with X-linked agammaglobulinemia (XLA). B.2 Dashboard output showing the most likely disease category (antibody deficiency) promoted to the top and risk calculation for the XLA patient. C.1 A case example of network information flow for a patient with chronic granulomatous disease (CGD) and associated findings. C.2 Dashboard output for the CGD patient displaying the most likely disease category (phagocyte disorder) promoted to the top with associated risk calculation. Note the arrows designating changes from the baseline probabilities as patient characteristics are entered.
(TIFF)
