# Supplementary figures and images for: PI Prob: A risk prediction and clinical guidance system for evaluating patients with recurrent infections
Source: PLoS One. 2021 Feb 16;16(2):e0237285. doi: 10.1371/journal.pone.0237285 (PMC7886140; doi:10.1371/journal.pone.0237285)

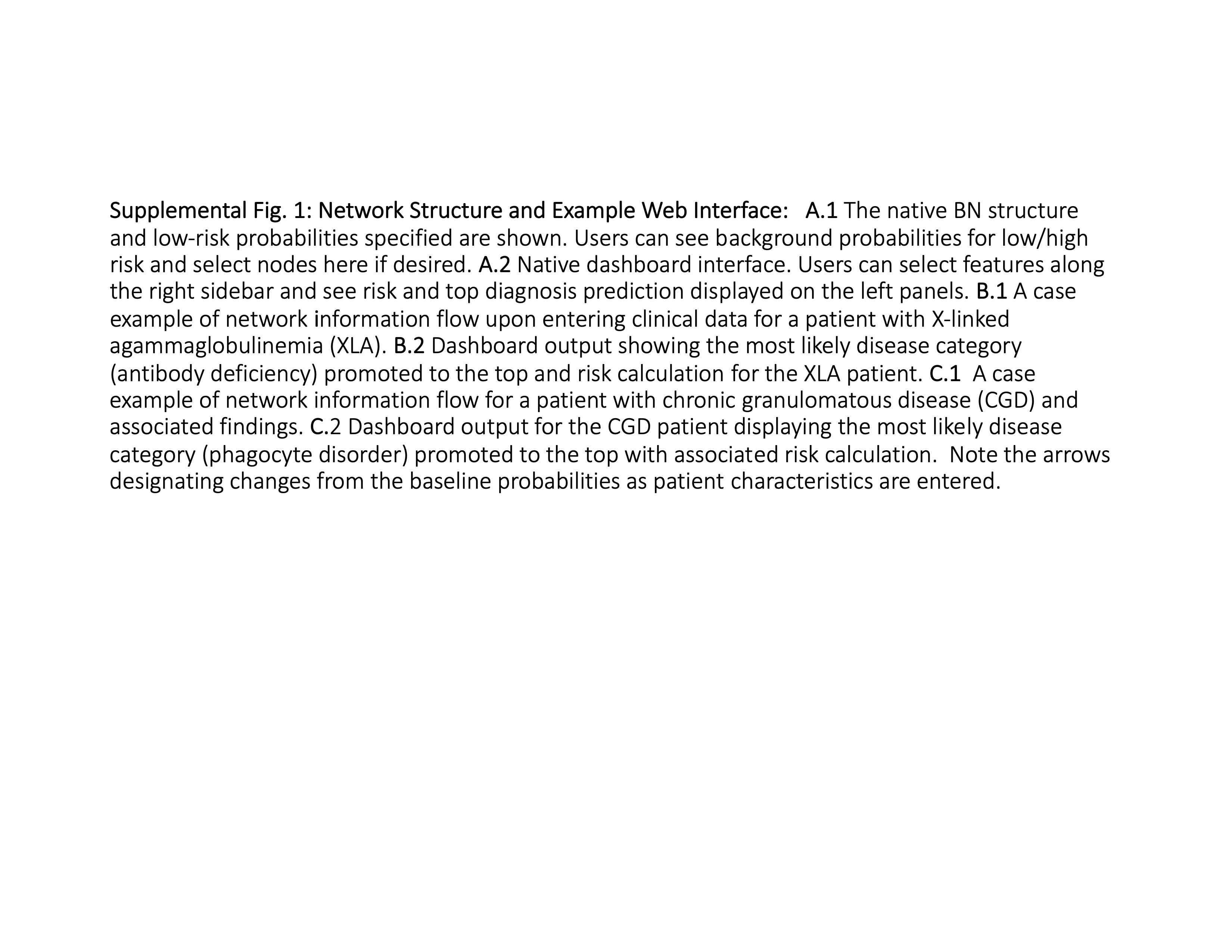

Supplement: S1 Fig — A.1 The native BN structure and low-risk probabilities specified are shown. Users can see background probabilities for low/high risk and select nodes here if desired. A.2 Native dashboard interface. Users can select features along the right sidebar and see risk and top diagnosis prediction displayed on the left panels. B.1 A case example of network information flow upon entering clinical data for a patient with X-linked agammaglobulinemia (XLA). B.2 Dashboard output showing the most likely disease category (antibody deficiency) promoted to the top and risk calculation for the XLA patient. C.1 A case example of network information flow for a patient with chronic granulomatous disease (CGD) and associated findings. C.2 Dashboard output for the CGD patient displaying the most likely disease category (phagocyte disorder) promoted to the top with associated risk calculation. Note the arrows designating changes from the baseline probabilities as patient characteristics are entered. (TIFF) [file pone.0237285.s001.tiff]
